# Supplementary material for: Pyruvate Dehydrogenase Kinase 1 inhibition mediated oxidative phosphorylation enhancement in cartilage promotes osteoarthritis progression
Source: BMC Musculoskelet Disord. 2023 Jul 20;24:597. doi: 10.1186/s12891-023-06585-6 (PMC10357736; doi:10.1186/s12891-023-06585-6)
Supplement: Supplementary file 1 — Additional file 1. [file 12891_2023_6585_MOESM1_ESM.zip › BP.Gsea.1653623667859/GOBP_BASE_EXCISION_REPAIR.html]

Details for gene set GOBP\_BASE\_EXCISION\_REPAIR[GSEA]

|  || Dataset | 55457\_collapsed\_to\_symbols.cls.cls #OA\_versus\_normal.cls.cls #OA\_versus\_normal\_repos |
| Phenotype | cls.cls#OA\_versus\_normal\_repos |
| Upregulated in class | OA |
| GeneSet | GOBP\_BASE\_EXCISION\_REPAIR |
| Enrichment Score (ES) | 0.3781839 |
| Normalized Enrichment Score (NES) | 1.4560188 |
| Nominal p-value | 0.03883495 |
| FDR q-value | 0.41768843 |
| FWER p-Value | 1.0 |
Table: GSEA Results Summary

  

Fig 1: Enrichment plot: GOBP\_BASE\_EXCISION\_REPAIR      
 Profile of the Running ES Score & Positions of GeneSet Members on the Rank Ordered List

  

| SYMBOL | TITLE | RANK IN GENE LIST | RANK METRIC SCORE | RUNNING ES | CORE ENRICHMENT || 1 | RPA3 | replication protein A3 [Source:HGNC Symbol;Acc:HGNC:10291] | 13 | 1.159 | 0.0746 | Yes |
| 2 | POLB | DNA polymerase beta [Source:HGNC Symbol;Acc:HGNC:9174] | 93 | 0.923 | 0.1286 | Yes |
| 3 | MPG | N-methylpurine DNA glycosylase [Source:HGNC Symbol;Acc:HGNC:7211] | 277 | 0.768 | 0.1644 | Yes |
| 4 | RPA2 | replication protein A2 [Source:HGNC Symbol;Acc:HGNC:10290] | 339 | 0.727 | 0.2071 | Yes |
| 5 | OGG1 | 8-oxoguanine DNA glycosylase [Source:HGNC Symbol;Acc:HGNC:8125] | 453 | 0.681 | 0.2427 | Yes |
| 6 | RPA1 | replication protein A1 [Source:HGNC Symbol;Acc:HGNC:10289] | 571 | 0.632 | 0.2748 | Yes |
| 7 | PARP2 | poly(ADP-ribose) polymerase 2 [Source:HGNC Symbol;Acc:HGNC:272] | 758 | 0.579 | 0.2980 | Yes |
| 8 | POLD1 | "DNA polymerase delta 1, catalytic subunit [Source:HGNC Symbol;Acc:HGNC:9175]" | 811 | 0.566 | 0.3309 | Yes |
| 9 | SMUG1 | single-strand-selective monofunctional uracil-DNA glycosylase 1 [Source:HGNC Symbol;Acc:HGNC:17148] | 1068 | 0.501 | 0.3437 | Yes |
| 10 | PCNA | proliferating cell nuclear antigen [Source:HGNC Symbol;Acc:HGNC:8729] | 1557 | 0.419 | 0.3331 | Yes |
| 11 | XPA | "XPA, DNA damage recognition and repair factor [Source:HGNC Symbol;Acc:HGNC:12814]" | 1615 | 0.411 | 0.3554 | Yes |
| 12 | PNKP | polynucleotide kinase 3'-phosphatase [Source:HGNC Symbol;Acc:HGNC:9154] | 1662 | 0.404 | 0.3782 | Yes |
| 13 | XRCC1 | X-ray repair cross complementing 1 [Source:HGNC Symbol;Acc:HGNC:12828] | 2182 | 0.340 | 0.3600 | No |
| 14 | LIG3 | DNA ligase 3 [Source:HGNC Symbol;Acc:HGNC:6600] | 2898 | 0.261 | 0.3214 | No |
| 15 | FAM168A | family with sequence similarity 168 member A [Source:HGNC Symbol;Acc:HGNC:28999] | 3143 | 0.235 | 0.3178 | No |
| 16 | NTHL1 | nth like DNA glycosylase 1 [Source:HGNC Symbol;Acc:HGNC:8028] | 3219 | 0.228 | 0.3269 | No |
| 17 | NEIL3 | nei like DNA glycosylase 3 [Source:HGNC Symbol;Acc:HGNC:24573] | 4006 | 0.156 | 0.2759 | No |
| 18 | POLQ | DNA polymerase theta [Source:HGNC Symbol;Acc:HGNC:9186] | 4293 | 0.129 | 0.2620 | No |
| 19 | ERCC5 | "ERCC excision repair 5, endonuclease [Source:HGNC Symbol;Acc:HGNC:3437]" | 4380 | 0.122 | 0.2633 | No |
| 20 | APEX2 | apurinic/apyrimidinic endodeoxyribonuclease 2 [Source:HGNC Symbol;Acc:HGNC:17889] | 4551 | 0.109 | 0.2572 | No |
| 21 | FEN1 | flap structure-specific endonuclease 1 [Source:HGNC Symbol;Acc:HGNC:3650] | 5212 | 0.056 | 0.2095 | No |
| 22 | MUTYH | mutY DNA glycosylase [Source:HGNC Symbol;Acc:HGNC:7527] | 5252 | 0.052 | 0.2099 | No |
| 23 | UNG | uracil DNA glycosylase [Source:HGNC Symbol;Acc:HGNC:12572] | 6078 | -0.011 | 0.1464 | No |
| 24 | POLL | DNA polymerase lambda [Source:HGNC Symbol;Acc:HGNC:9184] | 6214 | -0.022 | 0.1373 | No |
| 25 | HUWE1 | "HECT, UBA and WWE domain containing E3 ubiquitin protein ligase 1 [Source:HGNC Symbol;Acc:HGNC:30892]" | 6558 | -0.046 | 0.1137 | No |
| 26 | HMGA2 | high mobility group AT-hook 2 [Source:HGNC Symbol;Acc:HGNC:5009] | 6850 | -0.067 | 0.0954 | No |
| 27 | LIG1 | DNA ligase 1 [Source:HGNC Symbol;Acc:HGNC:6598] | 6944 | -0.075 | 0.0931 | No |
| 28 | ERCC6 | "ERCC excision repair 6, chromatin remodeling factor [Source:HGNC Symbol;Acc:HGNC:3438]" | 7939 | -0.150 | 0.0256 | No |
| 29 | SIRT6 | sirtuin 6 [Source:HGNC Symbol;Acc:HGNC:14934] | 8532 | -0.196 | -0.0076 | No |
| 30 | APEX1 | apurinic/apyrimidinic endodeoxyribonuclease 1 [Source:HGNC Symbol;Acc:HGNC:587] | 8767 | -0.213 | -0.0119 | No |
| 31 | WRN | WRN RecQ like helicase [Source:HGNC Symbol;Acc:HGNC:12791] | 10010 | -0.323 | -0.0875 | No |
| 32 | TDG | thymine DNA glycosylase [Source:HGNC Symbol;Acc:HGNC:11700] | 10728 | -0.396 | -0.1174 | No |
| 33 | NEIL1 | nei like DNA glycosylase 1 [Source:HGNC Symbol;Acc:HGNC:18448] | 10963 | -0.425 | -0.1078 | No |
| 34 | POLE | "DNA polymerase epsilon, catalytic subunit [Source:HGNC Symbol;Acc:HGNC:9177]" | 11026 | -0.433 | -0.0844 | No |
| 35 | MBD4 | "methyl-CpG binding domain 4, DNA glycosylase [Source:HGNC Symbol;Acc:HGNC:6919]" | 11114 | -0.445 | -0.0622 | No |
| 36 | RPS3 | ribosomal protein S3 [Source:HGNC Symbol;Acc:HGNC:10420] | 11245 | -0.463 | -0.0421 | No |
| 37 | PARG | poly(ADP-ribose) glycohydrolase [Source:HGNC Symbol;Acc:HGNC:8605] | 11786 | -0.547 | -0.0484 | No |
| 38 | DNA2 | DNA replication helicase/nuclease 2 [Source:HGNC Symbol;Acc:HGNC:2939] | 12148 | -0.631 | -0.0354 | No |
| 39 | POLG | "DNA polymerase gamma, catalytic subunit [Source:HGNC Symbol;Acc:HGNC:9179]" | 12369 | -0.699 | -0.0070 | No |
| 40 | USP47 | ubiquitin specific peptidase 47 [Source:HGNC Symbol;Acc:HGNC:20076] | 12476 | -0.739 | 0.0330 | No |
Table: GSEA details [plain text format]

  

Fig 2: GOBP\_BASE\_EXCISION\_REPAIR      
 Blue-Pink O' Gram in the Space of the Analyzed GeneSet

  

Fig 3: GOBP\_BASE\_EXCISION\_REPAIR: Random ES distribution      
 Gene set null distribution of ES for **GOBP\_BASE\_EXCISION\_REPAIR**
